# Characterization of humoral and cellular immunologic responses to an mRNA-based human cytomegalovirus vaccine from a phase 1 trial of healthy adults

**DOI:** 10.1128/jvi.01603-23

**Published:** 2024-03-25

**Authors:** Kai Wu, Yixuan Jacob Hou, Dan Makrinos, Runxia Liu, Alex Zhu, Matthew Koch, Wen-Han Yu, Yamuna D. Paila, Sumana Chandramouli, Lori Panther, Carole Henry, Anthony DiPiazza, Andrea Carfi

**Affiliations:** 1 Infectious Disease Research, Moderna, Inc., Cambridge, Massachusetts, USA; 2 Infectious Disease Development, Moderna, Inc., Cambridge, Massachusetts, USA; The University of Arizona, Tucson, Arizona, USA

**Keywords:** cytomegalovirus, immune response, messenger RNA, vaccine

## Abstract

mRNA-1647 is an investigational mRNA-based vaccine against cytomegalovirus (CMV) that contains sequences encoding the CMV proteins glycoprotein B and pentamer. Humoral and cellular immune responses were evaluated in blood samples collected from healthy CMV-seropositive and CMV-seronegative adults who participated in a phase 1 trial of a three-dose series of mRNA-1647 (NCT03382405). Neutralizing antibody (nAb) titers against fibroblast and epithelial cell infection in sera from CMV-seronegative mRNA-1647 recipients were higher than those in sera from control CMV-seropositive samples and remained elevated up to 12 months after dose 3. nAb responses elicited by mRNA-1647 were comparable across 14 human CMV (HCMV) strains. Frequencies of antigen-specific memory B cells increased in CMV-seropositive and CMV-seronegative participants after each mRNA-1647 dose and remained elevated for up to 6 months after dose 3. mRNA-1647 elicited robust increases in frequencies and polyfunctionality of CD4^+^ T helper type 1 and effector CD8^+^ T cells in samples from CMV-seronegative and CMV-seropositive participants after stimulation with HCMV-specific peptides. The administration of three doses of mRNA-1647 to healthy adults elicited high nAb titers with wide-breadth, long-lasting memory B cells, and strong polyfunctional T-cell responses. These findings support further clinical development of the mRNA-1647 vaccine against CMV.

**IMPORTANCE:**

Cytomegalovirus (CMV), a common virus that can infect people of all ages, may lead to serious health problems in unborn babies and those with a weakened immune system. Currently, there is no approved vaccine available to prevent CMV infection; however, the investigational messenger RNA (mRNA)–based CMV vaccine, mRNA-1647, is undergoing evaluation in clinical trials. The current analysis examined samples from a phase 1 trial of mRNA-1647 in healthy adults to better understand how the immune system reacts to vaccination. Three doses of mRNA-1647 produced a long-lasting immune response, thus supporting further investigation of the vaccine in the prevention of CMV infection.

**CLINICAL TRIALS:**

Registered at ClinicalTrials.gov (NCT03382405).

## INTRODUCTION

Cytomegalovirus (CMV) is a ubiquitous prototype pathogen from the β-herpesviruses family, with estimated prevalence of 60% and 90% in developed and developing countries, respectively (1, 2). Primary infection in healthy individuals usually is asymptomatic or causes mild mononucleosis-like symptoms. However, CMV infection may lead to serious complications in immunocompromised individuals, such as transplant recipients, or when fetuses are infected *in utero* (3). In transplant recipients, CMV infection can increase the risk of graft rejections, coinfection, or death and is the most common type of post-transplant viral infection (4, 5). Vertical transmission of CMV from a pregnant person to their fetus can cause congenital CMV infection (cCMV), which may lead to low birth weight, preterm birth, or neonatal death, as well as lifelong sequelae such as hearing loss or neurodevelopmental delays (6). CMV carries a high health and economic burden and is the most common infectious cause of birth defects in the United States, and the development of a prophylactic vaccine is a high public health priority (5–7).

To date, no vaccines against CMV have been approved, and past candidates showed limited success in clinical trials (5). Several vaccines are currently in development including mRNA-1647, an mRNA–based vaccine that is undergoing evaluation in phase 2 and 3 trials. mRNA-1647 contains sequences for two human CMV (HCMV) antigens, glycoprotein B (gB) and pentameric gH/gL/UL128/UL130/UL131A glycoprotein complex (pentamer), which target distinct cell entry processes during CMV infection (8, 9). Both gB and pentamer are essential for infection with CMV: gB mediates membrane fusion in all cell types, whereas pentamer primarily mediates receptor binding in epithelial cells, endothelial cells, and leukocytes, and minimally in fibroblasts (10–13). The double antigen design differentiates mRNA-1647 from the previous CMV vaccine approaches, many of which only targeted the gB protein (5). Previously, we demonstrated that pentamer, with correct conformation, was produced after mRNA delivery into mammalian cells by using a number of monoclonal antibodies targeting different epitopes (8). Other key advantages of an mRNA-based vaccine include the capacity to induce endogenous production of structurally intact protein antigens that mimic natural infection, as well as the scalability and flexibility of the vaccine platform, which allow rapid production and modification of the antigen sequence (14).

Previous studies of mRNA-based vaccines have shown that they can elicit both antigen-specific humoral responses and long-term cellular immune responses that are comparable to natural infection (15). For CMV, despite a multitude of studies on the roles of B-cell– and T-cell–mediated immunity (5, 16), the mechanistic correlates of protection have yet to be defined. Therefore, it’s desirable for a CMV vaccine to elicit robust levels of both humoral and cellular immune response.

This exploratory analysis evaluates cross-strain neutralization, memory B-cell, and multi-dimensional T-cell phenotypes in healthy adults following receipt of mRNA-1647 in a phase 1 first-in-human trial (NCT03382405). Our results demonstrate the robust humoral and cellular immune responses elicited by mRNA-1647, which often surpass those induced by natural infection.

## RESULTS

### Duration and breadth of neutralizing antibody response in seronegative participants after mRNA-1647 vaccination

**Fig 1:**
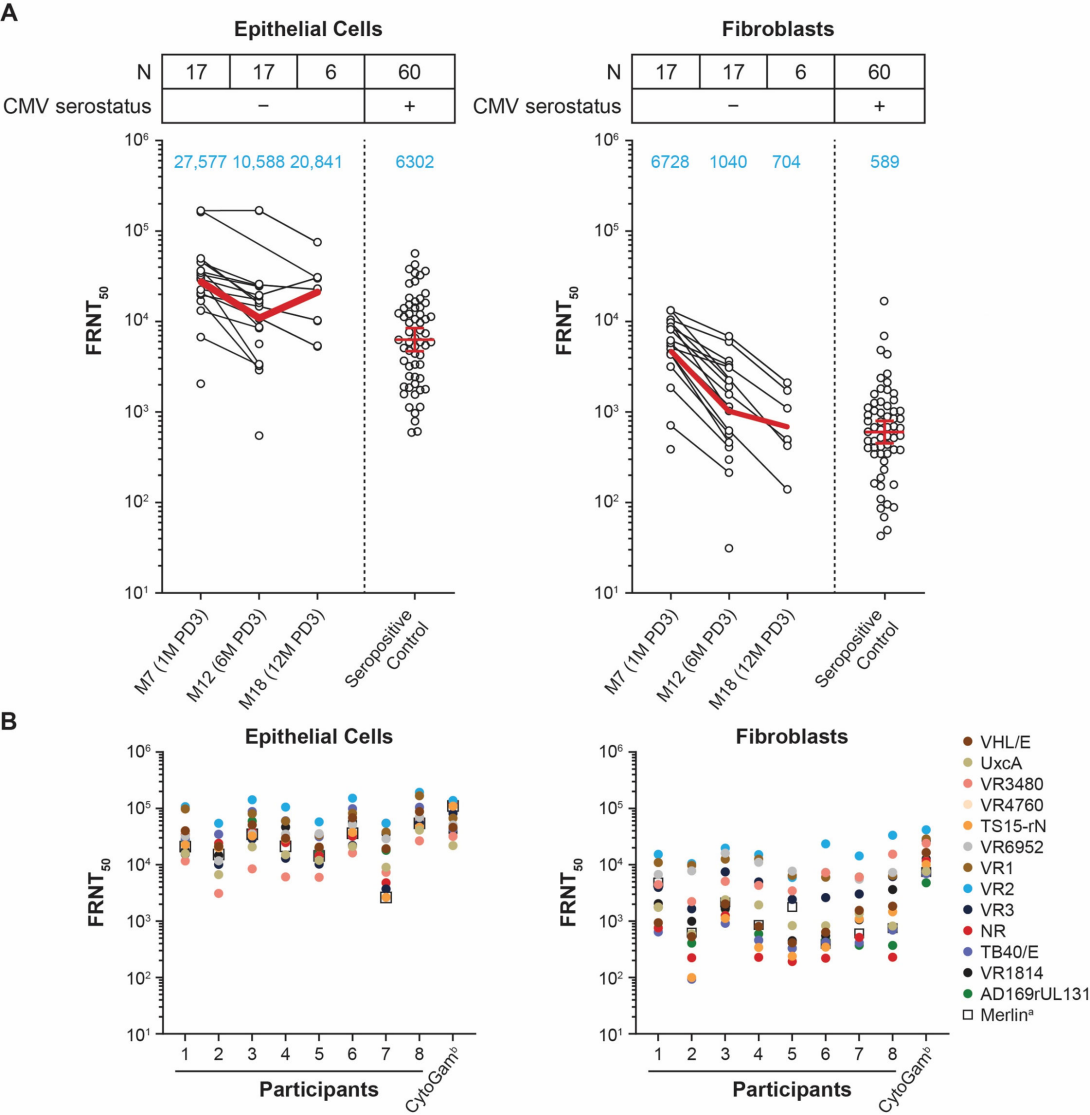
Level and breadth of nAb activity in seronegative participants after mRNA-1647 vaccination. (**A**) Neutralization of HCMV in epithelial cells and fibroblasts. Black dots indicate individual values and red lines indicate the geometric mean values across the timepoints (also indicated in the blue text). (**B**) nAb response against 14 HCMV strains in eight seronegative participants at month 12 (6 months PD3). The assay in fibroblasts was performed in the presence of 1.56% rabbit complement. ^a^nAb titers against the homologous vaccine strain Merlin. ^b^CMV hyperimmune globulin. CMV, cytomegalovirus; FRNT_50_, foci-reduction neutralization test with a 50% neutralization cutoff; HCMV, human CMV; M, month; PD, post-dose.

To assess the level of neutralizing antibodies (nAbs) elicited by mRNA-1647, we evaluated samples from 17 seronegative participants who received mRNA-1647 and compared the level to that of control samples from 60 seropositive individuals who were not vaccinated. The geometric mean titer of nAbs at month 7 (1 month post-dose 3 [PD3]) from vaccinated participants was approximately four-fold higher than that from control samples in epithelial cells and 11-fold higher in fibroblasts (Fig. 1A). nAb titers remained at a high level through month 18 (12 months PD3), but the sample size was small at this time point (*n* = 6) due to sample availability. Additionally, nAbs were also measured in fibroblasts without complement, where a decrease in nAb titers was observed compared with those with complement. However, the kinetics of nAbs in fibroblasts was similar between the two assay conditions (Fig. S1).

Given the limited breadth of neutralization of antibodies elicited by a previous CMV vaccine candidate, gB/MF59 (17), nAb breadth after vaccination with mRNA-1647 against 14 different HCMV strains was evaluated from seronegative participants at month 12 (6 months PD3). Compared with the Merlin strain, which mRNA-1647 is based on, the ranges of percent sequence similarity for each of the six antigens in the other 13 HCMV strains were: gB (93.51%–100%), gH (95.96%–99.72%), gL (97.84%–99.28%), UL128 (98.25%–99.42%), UL130 (97.66%–99.53%), and UL131A (99.22%–100%) (Table S1; Fig. S2). Compared with the nAb titers against the matched Merlin strain in epithelial cells, GMTs against other HCMV strains were similar or higher among all tested samples (Fig. 1B). This broad antibody neutralization response elicited by mRNA-1647 was comparable to the HCMV hyperimmune globulin (CytoGam) (Fig. 1B). In fibroblasts, nAb titers against many HCMV strains were also of similar or higher magnitude than those of the matched Merlin strain, while the range of nAb titers against different strains was wider than that in epithelial cells (Fig. 1B). These data suggest that mRNA-1647 elicits broad nAb responses in both fibroblasts and epithelial cells.

**Fig 2:**
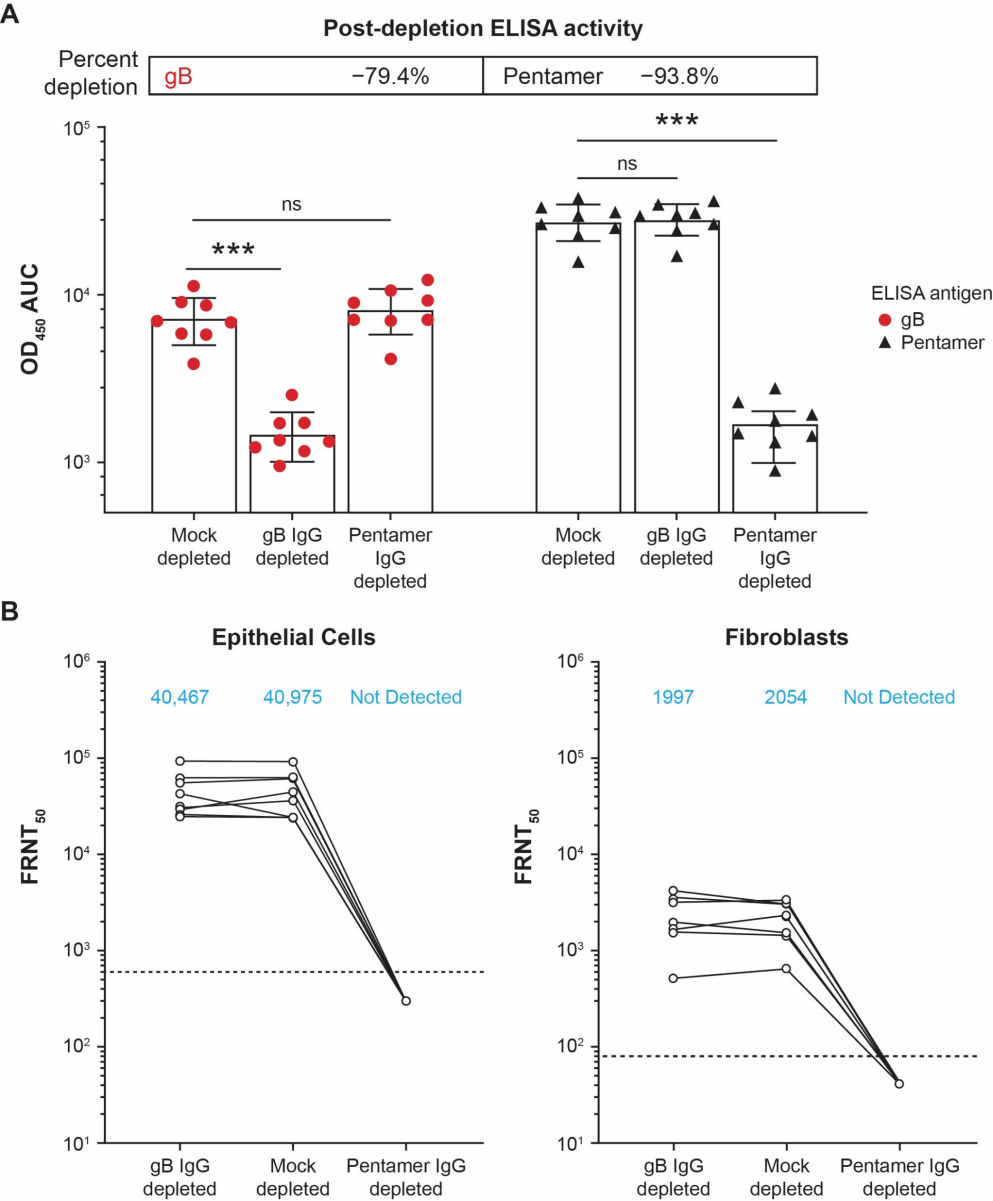
IgG depletion and nAb response in epithelial cells and fibroblasts from the sera of seronegative participants who received mRNA-1647. (**A**) gB-, pentamer-, and mock-IgG depletion efficiency in the sera of eight seronegative participants at month 7 (1 month PD3) as measured by ELISA. Statistical significance was calculated using the Mann-Whitney test. ***P < 0.001 vs mock-depleted cells. (**B**) nAb response of gB-, pentamer-, and mock-IgG–depleted sera against CMV infection in epithelial cells and fibroblasts. The assay in fibroblasts was performed in the presence of 1.56% rabbit complement. Blue text indicates geometric mean values. Dotted lines indicate the limit of detection. AUC, area under the curve; CMV, cytomegalovirus; ELISA, enzyme-linked immunosorbent assay, FRNT_50_, foci-reduction neutralization test with a 50% neutralization cutoff; gB, glycoprotein B; IgG, immunoglobulin G; nAb, neutralizing antibody; ns; not significant; OD_450_, optical density at 450 nm; PD, post-dose.

To determine the antigen specificity of nAbs, the immunoglobulin G (IgG) depletion of serum samples at month 7 (1 month PD3) was carried out using mock-, gB-, or pentamer-conjugated beads. The depletion efficiency was confirmed by enzyme-linked immunosorbent assay (ELISA) to be −79.4% for anti-gB IgG and −93.8% for anti-pentamer IgG (Fig. 2A). In anti-gB IgG–depleted sera of seronegative participants who received mRNA-1647, nAb response was similar to that of control sera with mock depletion for both epithelial cell and fibroblast infection (Fig. 2B). In contrast, anti-pentamer IgG-depleted sera had diminished neutralization potential in both cell types, suggesting the dominant role of anti-pentamer IgG in the neutralization response, regardless of cell type (Fig. 2B).

### Antigen-specific memory B-cell responses after mRNA-1647 vaccination in seronegative and seropositive participants

**Fig 3:**
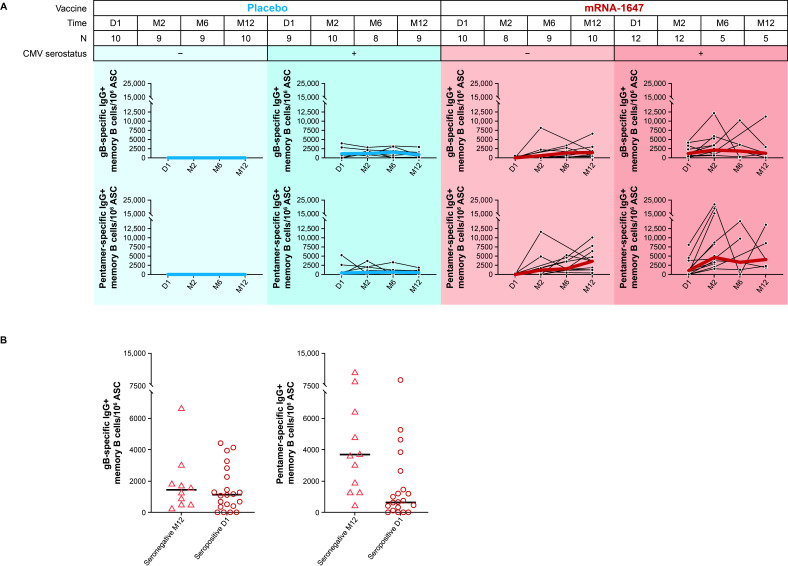
Frequencies of anti-gB and anti-pentamer IgG-secreting memory B cells in seronegative and seropositive participants who received mRNA-1647 or placebo. (**A**) Memory B-cell frequencies in seronegative and seropositive participants at day 1 (baseline), month 2 (2 months PD1), month 6 (4 months PD2), and month 12 (6 months PD3). Black lines indicate frequencies in individual samples, blue lines indicate the median frequencies in the placebo cohort, and red lines indicate the median frequencies in the mRNA-647 cohort. (**B**) Comparison of memory B-cell frequencies between seronegative participants post mRNA-1647 at month 12 (6 months PD3, *n* = 10) and all seropositive participants at day 1 (baseline, *n* = 21). Data displayed as individual values and medians. ASC, antibody-secreting cells; CMV, cytomegalovirus; D, day; gB, glycoprotein B; IgG, immunoglobulin G; M, month; MBC, memory B cells, PD, post-dose.

The frequencies of anti-gB and -pentamer IgG-secreting memory B cells were measured in blood samples from seronegative and seropositive participants collected at baseline, month 2 (2 months PD1), month 6 (4 months PD2), and month 12 (6 months PD3) after receiving mRNA-1647 or placebo. As expected, across both treatment groups, the frequency of anti-gB and -pentamer IgG-secreting memory B cells at baseline was higher in seropositive participants compared with the seronegative participants (Fig. 3A). After the mRNA-1647 vaccination in seronegative participants, the median frequency of both gB- and pentamer-specific memory B cells increased after each dose and remained elevated for up to 12 months. In seropositive participants, increases over baseline were observed in PD1 and largely remained at the same level through month 12; although the sample sizes were smaller at month 6 and month 12 compared with baseline (Fig. 3A). For comparison, no increases in B-cell frequencies were observed in seronegative or seropositive participants who received placebo (Fig. 3A). Importantly, in participants who received mRNA-1647, the median frequencies of anti-pentamer IgG-secreting memory B cells in seronegative participants at month 12 were higher than the baseline levels in seropositive participants, and frequencies of anti-gB IgG-secreting memory B cells were similar between these two groups (Fig. 3B). These data suggest that mRNA-1647 generates strong and durable antigen-specific memory B-cell responses.

### T-cell responses after mRNA-1647 vaccination in seropositive and seronegative participants

**Fig 4:**
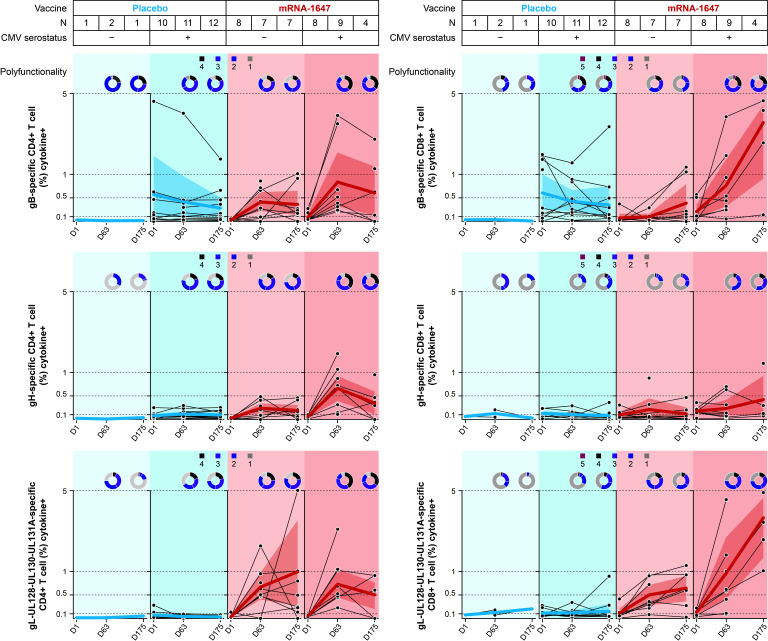
Antigen-specific T-cell response to the stimulation with gB, gH, and gL peptides in seronegative and seropositive participants who received mRNA-1647 or placebo. Antigen-specific CD4^+^ and CD8^+^ T-cell responses following *ex vivo* re-stimulation with gB, gH, and gL-UL128-UL130-UL131A peptides. Samples were collected at timepoints: day 1 (baseline), day 63 (1 week PD2), and day 175 (1 week PD3). Black lines indicate frequencies in individual samples; blue or red lines indicate the arithmetic mean frequencies across all samples. The polyfunctionality graphs represent the fraction of T cells expressing one to four functional markers (expression of cytokines or ligands) for CD4^+^ T cells and one to five functional markers for CD8^+^ T cells. The shade in the figure represents 95% CI across the patients in each treatment group. CMV, cytomegalovirus; D, day; gB, glycoprotein B; gH, glycoprotein H; gL, glycoprotein L; M, month; PD, post-dose.

The frequencies of the different T-cell populations and the cytokine or ligand production were quantified in blood samples from seronegative and seropositive participants collected at baseline, 1 week PD2, and 1 week PD3 in mRNA-1647 and placebo treatment groups. Antigen-specific CD4^+^ and CD8^+^ T cells were measured after the stimulation with three separate peptide pools: gB, gH, and gL-UL128-UL130-UL131A. Upon gB peptide stimulation, the frequencies of CD8^+^ and CD4^+^ T cells at 1 week PD3 (Day 175) of mRNA-1647 in seronegative participants approached or reached the same level as baseline in the seropositive placebo group (Fig. 4; Fig. S3). gL-UL128-UL130-UL131A stimulation resulted in even more pronounced CD4^+^ and CD8^+^ T-cell response, with frequencies much higher in the seronegative mRNA-1647 group than that of the seropositive placebo group across all the markers assessed, with the exception of interleukin-2 in CD8^+^ T cells (Fig. S4). Additionally, both gB- and gL-UL128-UL130-UL131A–specific responses in the mRNA-1647 group were further elevated in seropositive participants. In contrast, gH-specific CD8^+^ T-cell response was minimal or modest after mRNA-1647 immunization in seronegative and seropositive participants, and gH-specific CD8^+^ response at baseline was similar for seropositive participants receiving placebo or mRNA-1647 (Fig. 4; Fig. S5). However, mRNA-1647 elicited similar or higher gH-specific CD4^+^ T-cell response in both seronegative and seropositive participants as compared with the seropositive placebo group. The frequencies of IL-4^+^, IL-5^+^, and/or IL-13^+^ Th2 and IL-17A^+^ Th17 CD4^+^ T cells were not affected by the stimulation with any of the three peptide pools (Fig. S6).

Consistent with the results for the T-cell subpopulation frequencies, gB and gL-UL128-UL130-UL131A stimulation led to similar numbers of polyfunctional (simultaneously expressing ≥2 functional markers) CD4^+^ and CD8^+^ T cells in the seronegative mRNA-1647 group as in the seropositive placebo group, with lower similarity in CD8^+^ T cells with gL-UL128-UL130-UL131A (Fig. 4). Additionally, both gB- and gL-UL128-UL130-UL131A–specific increases in T-cell polyfunctionality were further elevated in seropositive participants. Overall, these data suggest that mRNA-1647 generated not only robust frequencies but also polyfunctional Th1-dominated CD4^+^ and effector CD8^+^ T-cell responses in both seronegative and seropositive participants.

## DISCUSSION

This article reports a comprehensive characterization of immune responses elicited by the investigational CMV vaccine mRNA-1647 in seropositive and seronegative healthy participants from the phase 1, randomized, first-in-human clinical trial. The current analysis demonstrated that three doses of mRNA-1647 (180 µg) induced robust and broad nAb responses against CMV infection in both epithelial cells and fibroblasts. Additionally, high levels of antigen-specific memory B cells remained detectable through 6 months after the last dose was administered. Furthermore, favorable Th1-biased CD4^+^ and CD8^+^ T-cell responses elicited by mRNA-1647 were similar to or higher than those observed from natural infection with CMV.

Neutralizing antibodies can play an important role in preventing CMV infection and disease (18), and early detection of pentamer-specific nAbs has been associated with lower rates of cCMV during primary infection (19). As CMV has a very broad cellular tropism, fibroblast and epithelial cells are the common cell types used to evaluate nAb responses to block different viral entry mechanisms (12, 20–22). In fibroblasts, entry is mainly mediated by the interaction of the viral ligand gH/gL/gO (trimer) and the cellular receptor platelet-derived growth factor receptor α (PDGFRα) (23–26). In contrast, viral entry into epithelial cells is mediated by the interaction of pentamer with neuropilin-2 or olfactory receptor family member OR14l1, as epithelial cells have undetectable levels of PDGFRα on the cell surface (27–29). During natural infection, nAbs can inhibit CMV infection in both fibroblasts and epithelial cells (30–34); however, the level of nAbs in epithelial cells is much higher due to the increased potency of pentamer-specific antibodies against the interaction of pentamer with its cellular receptor(s) in epithelial cells (35, 36).

In the gB/MF59 vaccine trial, low levels of nAbs were detected against epithelial cell infections (37); in fibroblasts, nAbs were detected at lower levels as observed during natural infection (17, 37) and were only capable of neutralizing the autologous strain in a cohort of postpartum participants (17). In another trial that evaluated a replication-defective CMV vaccine, nAb levels in both cell types at peak level were similar to those of CMV-seropositive controls and were stabilized at a lower level at 1-year post vaccination (38). In contrast, another gB-based vaccine trial demonstrated that the levels of gB-specific memory B cells were robust at 24 months, and nAbs in fibroblasts and CD4^+^ T cells were higher than in natural infection in vaccine recipients for up to 4 years (39). In this study, we observed that mRNA-1647 elicited higher nAb peak levels in both cell types in CMV-seronegative participants compared with CMV-seropositive controls. In addition to the standard neutralization assays using two laboratory strains (VR1814 and AD169), a panel of an additional 12 different HCMV strains, representing a wide genetic diversity, were used in the present study to test the cross-strain neutralizing activity in both cell types. The results demonstrated a broad neutralizing potential induced by mRNA-1647 against all tested strains in both epithelial cells and fibroblasts. Interestingly, nAb levels against many strains were higher than those against the vaccine-matched Merlin strain in both cell types. One possible explanation for this increase is that the abundance of pentamer or gH/gL/gO trimer on the virion can impact the susceptibility of CMV to nAbs (40). Recently, IgG binding to cell-associated gB was reported to correlate with protection against primary infection in the gB/MF59 vaccine trial (41). Additionally, maternal antiviral non-neutralizing antibodies, specifically those that mediate antibody-dependent cellular cytotoxicity and antibody-dependent cellular phagocytosis, have been shown to be associated with a reduced risk of *in utero* CMV transmission (42, 43). The characterization of cell-associated gB-specific antibody response and non-neutralizing functional antibodies is of particular interest and warrants further investigation.

The contribution of antigen-specific antibodies to neutralizing activity in fibroblasts and epithelial cells has been studied in sera of CMV-seropositive individuals and in hyperimmune globulin (HIG) from pooled human plasma with high anti-CMV titers (37, 44–46). In epithelial cells, pentamer-specific antibodies are the main drivers for neutralizing activity in both sera from CMV-seropositive individuals and HIG (45, 46), which is also the case for nAbs elicited by mRNA-1647, supported by the results reported here. In fibroblasts, anti-gB and -gH/gL antibodies have been described to exert nAb activity (37, 44, 45). Several studies of sera from seropositive individuals have shown that depletion with gB resulted in modest reduction of nAb in fibroblasts (37, 44, 45). In contrast, anti-gH/gL in HIG is the main contributor of neutralization activity in fibroblasts (46). This study shows that anti-pentamer antibodies contribute most of the nAb activity elicited by mRNA-1647 in fibroblasts. As UL128L is dispensable for CMV infection in fibroblasts (47), anti-gH/gL antibodies induced by mRNA-1647 likely contribute to the nAb activity in fibroblasts. Such contribution is consistent with the results from a depletion study in HIG samples in which anti-gH/gL dominated the nAb activity against CMV in fibroblasts (46). Taken together, a strong anti-pentamer antibody response translated into a robust nAb response in both fibroblasts and epithelial cells.

Following the initial exposure to antigens that leads to antibody production by short-lived plasma cells and germinal center B cells, the levels of antibodies are sustained by antigen-specific long-lived plasma cells and memory B cells (48–50). Upon exposure to pathogens, pre-existing memory B cells can be quickly activated and proliferate to produce antibodies with high affinity (50). While the measurement of long-lived plasma cells is difficult due to the challenges with sampling from bone marrow, memory B cells can be readily assessed in peripheral blood (51, 52). In our study, mRNA-1647 elicited high levels of antigen-specific memory B-cell response. Given the slow replication cycle of the CMV virus, the quick recall response by memory B cells with high frequencies can further inhibit viral infection in the early infection stage. Therefore, in addition to the robust production of nAbs, the increased frequencies of antigen-specific memory B cells elicited by mRNA-1647—compared with placebo—have a potential to further enhance the protection against CMV infection, spread, and transmission. In addition to a robust and broad humoral response, mRNA-1647 induced strong Th1-dominated CD4^+^ and CD8^+^ T-cell responses. Notably, gL-UL128-UL130-UL131A–specific CD4^+^ and CD8^+^ T-cell responses in seronegative samples were elevated compared with those from seropositive samples at baseline. This elevated response is consistent with the increased neutralizing activity in the epithelial cells, which likely results from strong expression and presentation of pentamer by mRNA-1647 (8). Importantly, a high proportion of the mRNA-1647–elicited T cells were polyfunctional. The strong T-cell responses, especially against pentamer, are notable, given that the components of pentamer are typically known as neutralizing targets, rather than major T-cell antigens like viral proteins pp65 and IE1 (53). This could result from the robust expression of pentamer as observed *in vitro* after mRNA delivery (8). In addition, CMV encodes multiple viral proteins (US2, US3, US6, and UL11) to downregulate major histocompatibility complex protein expression and preferentially present certain antigens to T cells (54). It is plausible that the components of pentamer are not well-presented during infection, but vaccination overcomes such a barrier in the absence of the immune-evasive mechanism during viral infection. Taken together, mRNA-1647 induces strong and polyfunctional CD4^+^ and CD8^+^ T-cell–mediated immunity.

The overall humoral and cellular immunity induced by the mRNA-1647 CMV vaccine candidate is consistent with that demonstrated by the mRNA-based COVID-19 vaccines (55, 56), extending the potential of this platform to DNA viruses. The strong humoral and cellular immunity elicited by mRNA-based vaccines demonstrates the potential of mRNA technology and makes mRNA-1647 an excellent vaccine candidate to prevent CMV infection and diseases. Limitations of this study, including a relatively small sample size and a single-dose group (180 µg), can be addressed in future studies with larger cohorts.

In conclusion, the administration of three 180 µg doses of mRNA-1647 to healthy adults elicited high titers of nAbs, a wide breadth of neutralization, and generation of long-lasting memory B cells and a strong polyfunctional T-cell response in CMV-seropositive and seronegative recipients. These findings support further clinical development of the mRNA-1647 vaccine.

## MATERIALS AND METHODS

### Study design

This phase 1, randomized, observer-blind, placebo-controlled, dose-ranging, first-in-human trial (NCT03382405) included healthy participants aged 18–49 years who were either CMV seronegative or CMV seropositive. Participants were randomly assigned to receive three injections of mRNA-1647 (30 µg, 90 µg, 180 µg, 300 µg) or placebo. The current exploratory analysis included participants from the trial who received the 180 µg dose of mRNA-1647. Study injections were administered at day 1, month 2, and month 6; follow-up visits were scheduled through 12 months after the third injection. A detailed description of the participants and sample collection for all assays is available in the Supplementary Methods and Fig. S7.

### Cells and viruses

A detailed description of the cell lines and viruses used in this study is available in the Supplementary Methods.

### Neutralizing antibody response assays

CMV nAb titers were determined using an IE-1 immunostaining procedure. Antigen-specific antibodies were depleted from human serum samples by incubating the serum with CMV antigen-coupled magnetic beads. Antibody-depleted serum samples were subsequently subjected to ELISA quantification of CMV gB and pentamer. Specific details for these assays are provided in the Supplementary Methods.

### B-cell and T-cell response assessments

Frequencies of antigen-specific memory B cells and phenotyping of antigen-specific T cells in response to HCMV peptide stimulation in peripheral blood mononuclear cells (PBMCs) were analyzed using ELISpot assays and flow cytometry, respectively. Specific details for these assays are provided in the Supplementary Methods.

### Statistics

Details of the statistical methods for each analysis are provided in the figure captions, as well as in the Supplementary Methods. Most statistical analyses were performed using Microsoft Excel and/or GraphPad Prism 9 (GraphPad Software Inc.); the T-cell polyfunction analyses were conducted using a customized script in Python.

## Data Availability

Upon request, and subject to review, Moderna, Inc. will provide the data that support the findings of this study. Subject to certain criteria, conditions, and exceptions, Moderna, Inc. may also provide access to related individual anonymized participant data.

## SUPPLEMENTAL MATERIAL

Supplemental material jvi.01603-23-s0001.docx Additional information pertaining to participants and assessments, cells and viruses, etc.

ASM does not own the copyrights to Supplemental Material that may be linked to, or accessed through, an article. The authors have granted ASM a non-exclusive, world-wide license to publish the Supplemental Material files. Please contact the corresponding author directly for reuse.
