# Supplementary material for: Characterization of humoral and cellular immunologic responses to an mRNA-based human cytomegalovirus vaccine from a phase 1 trial of healthy adults
Source: J Virol. 2024 Mar 25;98(4):e01603-23. doi: 10.1128/jvi.01603-23 (PMC11019844; doi:10.1128/jvi.01603-23)

# Supplementary Material

**Participants and Assessments**

Response and specificity of neutralizing antibodies (nAbs) were measured in the sera of cytomegalovirus (CMV)-seronegative participants who received three 180-μg doses of mRNA-1647. Blood samples for antibody testing were collected during visits at months 7, 12, and 18, corresponding to 1 month, 6 months, and 12 months post-dose 3 (PD3), respectively (**Supplementary Fig. 7**)**.** Control sera from seropositive individuals were obtained from commercial blood repositories (BioIVT, SeraCare, Innovative Research, Aalto Bio, and Discovery Life Sciences).

B-cell frequency was assessed in blood collected from CMV-seronegative and -seropositive participants who received three 180-μg doses of mRNA-1647 or placebo. Blood samples for these assessments were collected during visits at day 1 (baseline) and months 2, 6, and 12, corresponding to 2 months post-dose 1 (PD1), 4 months post-dose 2 (PD2), and 6 months PD3 (**Supplementary Fig. 7**). For the analysis of antibody-mediated immunogenicity, a volume of approximately 10 mL of whole blood was drawn at each time point. CMV-specific T-cell magnitude and quality (including polyfunctionality) were assessed in blood collected from CMV-seronegative and -seropositive participants who received three 180-μg doses of mRNA-1647 or placebo in dose selection phase B. Blood samples for these assessments were collected at day 1 (baseline), day 63 (1 week PD2), and day 175 (1 week PD3) (**Supplementary Fig. 7**). For analysis of cell-mediated immunity, a volume of approximately 48 mL of whole blood was drawn at each time point.

**Cells and Viruses**

HEL-299 (ATCC, cat. no. CCL-137) and ARPE-19 (ATCC, cat. no. CRL-2302) were cultured in Dulbecco’s Modified Eagle Medium (DMEM) supplemented with 10% fetal bovine serum (FBS) and DMEM/F12 1:1 mix supplemented with 10% FBS, respectively. The following viruses were reconstituted from bacterial artificial chromosome infectious clones by electroporation and one subsequent expansion in ARPE-19 cells: Merlin (pAL1120, gift of Richard Stanton), TB40/E and VHL/E (gifts of Christian Sinzger), AD169rUL131A (gift of Thomas Shenk), TS15-rN and UxcA (gifts of Michael McVoy). The following viruses were expanded in ARPE-19 from seed virus stocks: VR1814, VR1, VR2, VR3, VR3480, VR4760, VR6952 (gifts of Daniele Lilleri), and NR (gift of Hua Zhu).

**Neutralizing Antibody Response Assays**

*Cell-Based Neutralizing Assay*

Human serum samples were serially diluted 3-fold and pre-incubated at a 1:1 ratio with corresponding strains of human CMV (HCMV) targeting 1000 focus-forming units (FFU)/well in 96-well U-bottom plates (Corning, cat. no. 3799). For fibroblast assays containing complement, baby rabbit complement (Cedarlane, cat. no. CL3441-s) was thawed fresh and diluted with the virus mixture to achieve a final concentration of 1.56%. After 1 hour incubation at 37°C, 100 μL of the virus/serum mixture was transferred to 96-well plates which were seeded the prior day at 20,000 cells/well with either HEL-299 in gelatin-coated plates (Corning, cat. no. 356689) or ARPE-19 in tissue culture (TC)-treated plates (Corning, cat. no. 3598). Plates were fixed with a 1:1 mixture of methanol/ethanol at 18 hours post infection. Cells were then washed once with phosphate-buffered saline (PBS) with 0.05% Tween (PBST) and subsequently incubated at room temperature for 2 hours with a primary mouse-anti-human IE-1–specific monoclonal antibody (mAb) (Millipore, cat. no. MAB810-500UG), then washed with PBST and incubated for 1 hour at room temperature with a horseradish peroxidase (HRP)-conjugated goat–anti-mouse secondary mAb (Jackson Immunoresearch, cat. No.115-035-166). A signal was detected with TrueBlue Peroxidase Substrate (KLP, cat. no. 5510-0030), imaged, and quantified using an Immunospot CTL Analyzer.

*Antibody Depletion*

His-tagged HCMV glycoprotein B (gB) and pentamer antigens were separately coupled to HisPur™ Nickel-NTA Magnetic Beads (ThermoFisher, cat. no. 88831) that had been prepared according to manufacturer recommendations. An irrelevant protein, EBV gp42, was coupled to a separate batch of beads to serve as mock control. Next, human serum samples diluted 1:10 in PBS were mixed with the antigen-coupled beads and incubated at room temperature with end-to-end rotation. After 20 minutes of incubation, beads were removed from the sample with DynaMag™-2 Magnet (Invitrogen, cat. no. 12321D). Four rounds of depletion with fresh beads were performed for each serum sample. Following the final round of depletion, all magnetic beads were eliminated from the samples. Finally, the serum samples were sterile filtered using 0.22-μm pore size filters (Corning, cat. no. CLS8160).

*Antibody Quantification by Enzyme-Linked Immunosorbent Assay (ELISA)*

HCMV gB and pentamer were coated separately on MaxiSorp 96-well flat-bottom ELISA plates (Nunc, cat. no. 439454) and stored at 4°C overnight. Coated plates were blocked with 200 μL of SuperBlock™ Blocking Buffer (ThermoFisher, cat. no. 37515) per well and incubated at 37°C for 1.5 hours. Coated and blocked plates were then washed 4 times with PBS + 0.05% Tween-20 using a BioTek BioStack Microplate Washer. Mock-depleted, gB-depleted, and pentamer-depleted serum samples were diluted 1:100 in PBS and subsequently diluted 3-fold for a total of 8 serial dilution steps. Diluted serum samples were transferred to the prepared ELISA plates and then incubated at 37°C for 2 hours. After washing plates 4 times with PBST, a 1:10,000 diluted anti-human IgG secondary antibody conjugated to HRP was transferred to the plates and incubated at 37°C for 1 hour. After washing plates 4 times with PBST, 100 μL of room temperature 3,3’, 5,5”-tetramethylbenzidine (TMB) substrate was transferred to the plates for 7.5 minutes then quenched with 100 μL of KPL TMB Stop Solution (SeraCare, catalog number 5150-0021). Absorbance was measured at 450 nm using a spectrophotometer (Molecular Devices SpectraMax ABS Microplate Reader). Half-maximal effective concentration (EC_50_) values were calculated in GraphPad Prism using the log(inhibitor) versus response-variable slope function for nonlinear regression (4-parameter curve).

**B-Cell and T-Cell Response Assessments**

*Memory B-Cell ELISpot Assay*

Peripheral blood mononuclear cells (PBMCs) were thawed in ELISpot media (Roswell Park Memorial Institute [RPMI]) supplemented with 10% heat-inactivated FBS, 1´ penicillin-streptomycin, 2 mM of L-glutamine, 10 mM Hepes, 1 mM sodium pyruvate, and 50 μM 2-mercaptoethanol). After washing, cells were resuspended into complete media containing 6 μg/mL CpG-ODN (InvivoGen, cat. no. tlrl-2006), 10 ng/ml lectin pokeweed mitogen (Sigma, cat. no. L9379), and protein A from *Staphylococcus aureus* (Sigma, cat. no. P7155) diluted at 1:10,000. Cells were plated in a 24-well plate with 1´10^6^ PBMCs per well and stimulated for 5 days at 37°C in a 5% CO_2_ incubator. The 96-well polyvinylidene difluoride (PVDF) membrane plate (Millipore, MSIPS4510) was pre-wetted with 35% ethanol and coated with 5 μg/mL recombinant HCMV glycoprotein B (gB) or pentamer antigens. To detect all immunoglobulin G (IgG)-secreting cells, 1 column of the plate was coated with 5 μg/mL goat anti-human Ig (KPL, cat. no. 01-10-07) instead of the recombinant antigens. The plate was incubated at 4°C for at least 18 hours. Plate was washed with 1´ PBS and blocked with ELISpot media for 3 hours at 37°C prior to use. After 5 days of stimulation, cells were collected from the 24-well culture plate, washed, and resuspended into ELISpot media with 5´10^6^ cells/mL. Cells were plated onto the ELISpot plates with 1´10^6^ cells as a starting point. Cells were serially diluted 2-fold in ELISpot media to capture the countable spots in multiple wells. For total Ig wells, cells were plated starting at 0.1´10^6^ cells/well, followed by 2-fold serial dilution. After the cells were seeded, the plate was incubated for 12-18 hours in a 5% CO_2_ incubator at 37°C. The plate was then washed with PBST and incubated with anti-human IgG-biotin (Southern Biotechnology, cat. no. 2040-08) diluted 1:1000 in PBST + 1% FBS for 2.5 hours at room temperature . After incubation with the primary antibody, the plate was washed and incubated with streptavidin-AP (Southern Biotechnology, cat. no. 7100-04) diluted 1:500 in PBST + 1% FBS. Lastly, the plate was developed using nitro-blue tetrazolium/5-bromo-4chloro-3’-indolyphosphate (NBT/BCIP) (Thermo Fisher Scientific, cat. no. 34-042). Data are represented as the frequency of antigen-specific IgG^+^ B cells per million of total IgG^+^ memory B cells.

*Human Intracellular Cytokine T-Cell Assay*

Frozen PBMCs were quickly thawed in a water bath at 37°C and immediately transferred to prewarmed culture media (RPMI 1640 medium supplemented with L-glutamine, penicillin/streptomycin antibiotic, and 5% heat-inactivated (HI) human AB serum [Gibco, cat. no. A1049101; Gibco, cat. no. 15140-122; Sigma, cat. no. H3667]) containing 50 U/mL benzonase nuclease (EMD Millipore, cat. no. 71206-3). After subsequent washes, PBMCs were counted, seeded at 2´10^6^ cells/well, and rested overnight at 37°C in a 5% CO_2_ incubator.

The next day, cells were washed with culture media and incubated with 0.5 µg/mL of αCD40 antibody (Miltenyi, cat. no. 130-094-133) for 15 minutes at 37°C. Subsequently, a stimulation cocktail containing 2 µg/mL of HCMV peptide pools (gB, gH, or gL-UL128-UL130-UL131A) , CD107a (Clone: H4A3; PE), NA/LE Fc Block (BD, cat. no. 564765), and protein transport inhibitor containing brefeldin A and monensin (ThermoFisher, cat. no. 00-4980-03) in culture media was overlayed. Control cells were incubated with peptide pool–matched quantity of dimethylsulfoxide (DMSO) (0.5% final concentration) as a no-peptide stimulation control or PMA/ionomycin (ThermoFisher, cat. no. 00-4970-03). Cells were incubated for 7.5 hours at 37°C in a 5% CO_2_ incubator and then stored overnight at 4°C. Cells were washed in 1´ PBS and stained in fixable live-dead stain in UV Blue (Invitrogen, cat. no. L23105) for 15 minutes at room temperature. Cells were washed once in flow cytometry buffer (FC buffer, 1´ PBS, 2% HI-FBS [Gibco, cat. No. 10082-147], and 0.05% sodium azide [VWR, cat. no. BDH7465-2]), then resuspended in 100 µL/well of surface markers antibody cocktail diluted in Brilliant Stain Buffer (BD, cat. no. 566385) for 15 minutes at room temperature and protected from light. Cells were washed in FC buffer, then fixed and permeabilized in fixation/permeabilization solution for 20 minutes at 4°C (BD Biosciences, cat. no. 554714). After washing in 1´ Perm/Wash buffer (containing 1´ FBS, 0.1% saponin, and 0.009% sodium azide in distilled water), cells were resuspended in 100 µL of intracellular markers antibody cocktail diluted in Brilliant Stain Buffer and Perm/Wash solution for 30 minutes at 4°C and protected from light. Finally, cells were washed twice in 1´ Perm/Wash buffer, then resuspended in FC buffer containing 0.5% paraformaldehyde before filtering through a 96-well plate using a 30-µm filter (Pall Corporation) prior to running on a 5-laser Aurora Spectral Flow Cytometer (Cytek Biosciences). Data analysis was performed using FlowJo version 10.8.1 (Treestar Inc.), Microsoft Excel, and GraphPad Prism 9 FlowAI was applied to all samples to remove irregular events. Cells were then gated on lymphocytes, singlets, live, CD3^+^, non-myeloid/B cell/NK (CD14, CD16, CD19, and CD56), then into populations of CD8^+^,CD4^-^ or CD8^-^,CD4^+^ T cells. Naïve cells CD45RA^+^, CCR7^+^ were removed (**Supplementary Fig. 8**). For cytokine analysis, CD4 subsets were gated on CD69^+^ and split into 4 subsets: Th1: IFNγ^+^, TNFα^+^, IL-2^+^; Th2: IL-4^+^, IL-5^+^, IL-13^+^; Th17: IL-17A^+^; and CD40L^+^. CD8 subsets were gated on CD69^+^ and effector markers: IFNγ^+^, TNFα^+^, IL-2^+^, CD107a^+^ and granzyme B. All antibodies are listed in **Supplementary Table 2**. To determine specific responses to peptide pools, percent positive (%) values were subtracted from DMSO controls for each sample. A threshold of 0 was applied to all samples to evaluate positive responders [1]. Combination Boolean gating was applied in FlowJo to assess CD4^+^ T cells for CD40L^+^, IFNγ^+^, TNFα^+^, and/or IL-2^+^ polyfunctionality. For CD8^+^ polyfunctionality IFNγ^+^, TNFα^+^, IL-2^+^, CD107a^+^, and/or granzyme B were assessed. Any cell positive for ≥1 of the following mediators (in conjunction with CD69) was considered antigen-specific; CD4^+^ T cells: IFNγ, TNFα, IL-2, CD40L; CD8^+^ T cells: IFNγ, TNFα, IL-2, CD107a, granzyme B. The sum of mediators with the same number of functions for each sample were then averaged across all participants within the same group and depicted as donut charts above each line graph representing the frequency of antigen-specific T-cell responses over time.

**References**

1. da Silva Antunes R, Pallikkuth S, Williams E, Dawen Yu E, Mateus J, Quiambao L, Wang E, Rawlings SA, Stadlbauer D, Jiang K, Amanat F, Arnold D, Andrews D, Fuego I, Dan JM, Grifoni A, Weiskopf D, Krammer F, Crotty S, Hoffer ME, Pahwa SG, Sette A. 2021. Differential T-Cell Reactivity to Endemic Coronaviruses and SARS-CoV-2 in Community and Health Care Workers. J Infect Dis. 224(1):70-80.

## Supplementary Table 1. Amino acid homology of 13 HCMV strains compared with Merlin

|  | |
| --- | --- |
| **Protein** | **Amino acid homology range, %** |
| gB | 93.51-100 |
| gH | 95.96-99.72 |
| gL | 97.84-99.28 |
| pUL128 | 98.25-99.42 |
| pUL130 | 97.66-99.53 |
| pUL131A | 99.22-100 |

## Supplementary Table 2. List of antibodies used in flow cytometry

| **Target** | **Clone** | **Color** | **Cat. no.** | **Manufacturer** | **Dilution** |
| --- | --- | --- | --- | --- | --- |
| Live/Dead | NA | UV Blue | L23105 | ThermoFisher | 0.125:100 |
| CD3 | SK7 | BUV496 | 741206 | BD | 1.25:100 |
| CD14 | 63D3 | AF488 | 367130 | Biolegend | 0.3:100 |
| CD16 | 3G8 | AF488 | 302019 | Biolegend | 0.04:100 |
| CD19 | HIB19 | AF488 | 302219 | BD | 0.2:100 |
| CD56 | 5.1H11 | AF488 | 362518 | Biolegend | 0.3:100 |
| CD8 | SK1 | BUV805 | 612890 | BD | 0.6:100 |
| CD4 | SK3 | C Fluor B548 | R7-20043 | Cytek Biosciences | 1.25:100 |
| CD45A | HI100 | BUV395 | 740298 | BD | 0.3:100 |
| CCR7 | 150503 | PE-CF 594 | 562381 | BD | 5:100 |
| CD69 | FN50 | APC-Fire 750 | 310946 | Biolegend | 0.2:100 |
| IFNɣ | B27 | BV480 | 566100 | BD | 1.25:100 |
| TNFα | Mab11 | BV750 | 566359 | BD | 5:100 |
| IL-2 | MQ1-17H12 | AF647 | 500315 | Biolegend | 2.5:100 |
| IL-4 | MP4-25D2 | BV786 | 564113 | BD | 2.5:100 |
| IL-5 | TRFK5 | BV421 | 504311 | Biolegend | 2.5:100 |
| IL-13 | JES10-5A2 | PE-Cy7 | 501914 | Biolegend | 0.6:100 |
| IL-17A | BL168 | BV605 | 512326 | Biolegend | 2.5:100 |
| CD40L | 24-31 | PacificBlue | 310820 | Biolegend | 0.6:100 |
| CD107a | H4A3 | PE | 328608 | Biolegend | 0.08:100 |
| Granzyme B | GB11 | R718 | 566964 | BD | 0.6:100 |

## Supplementary Fig 1. Neutralization of CMV in fibroblasts in presence or absence of complement.

CMV, cytomegalovirus; D, day; FRNT50, foci-reduction neutralization test with a 50% neutralization cutoff; LOD, limit of detection; M, month; PD, post-dose.


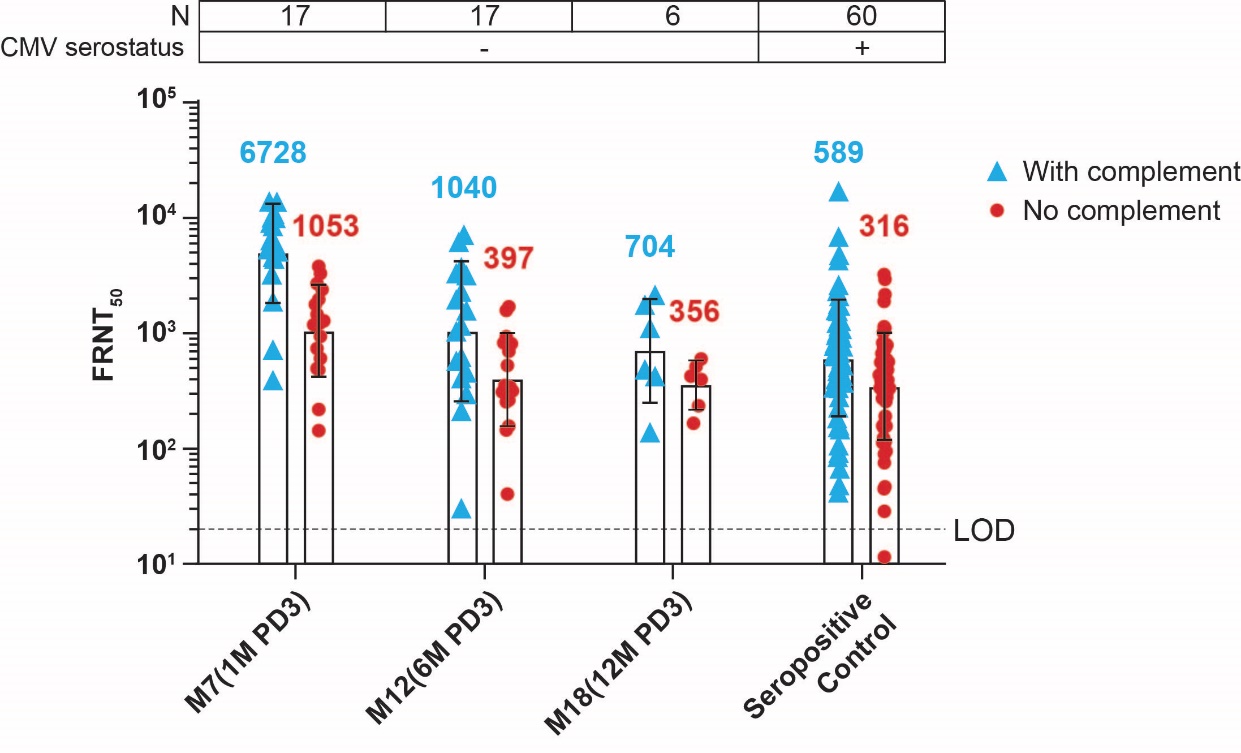


## **Supplementary Fig 2.** Genetic diversity of antigens used in mRNA-1647 components. Phylogenetic analysis of the gB, gH, gL, UL128, UL130, and UL131A proteins from global HCMV strains. The strains used in the cross-neutralization assay are highlighted in red and the Merlin strain used for mRNA-1647 design is highlighted in blue. The protein sequences of all other strains (N >370) were downloaded from GenBank. The phylogenetic trees were generated using Geneious Prime software, using the neighbor-joining method. gB, glycoprotein B; gH, glycoprotein H; gL, glycoprotein L; HCMV, human cytomegalovirus.


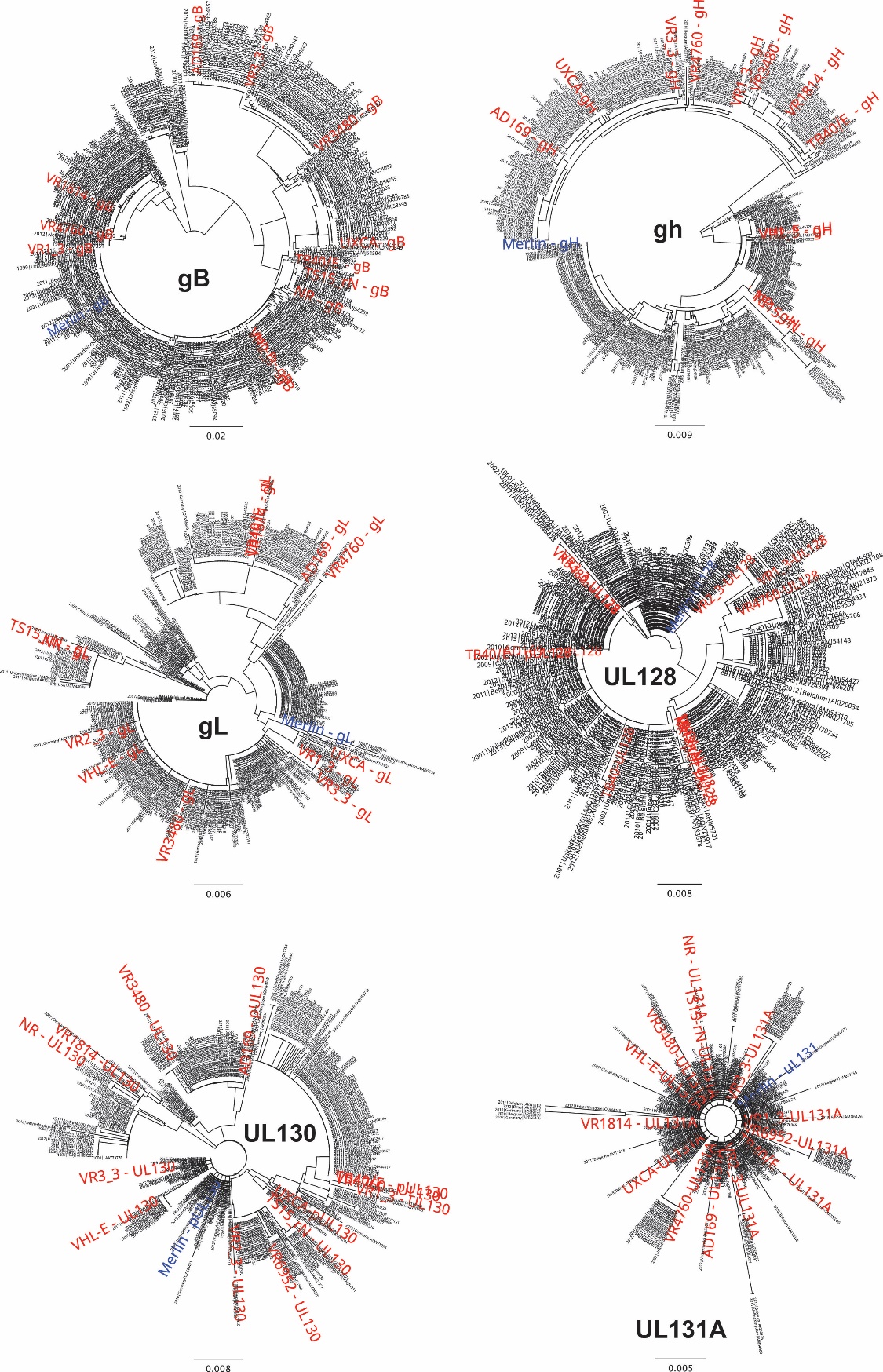


## Supplementary Fig 3. T-cell response in seronegative and seropositive participants who received mRNA-1647 or placebo to stimulation with gB peptide. (A) Frequencies of different CD4^+^ T-cell populations after stimulation with gB peptide. The following markers were measured: CD40L, IL-2, TNFα, IFNγ. (B) Frequencies of CD8^+^ T cells after stimulation with gB peptide. The following effector cell markers were measured: CD107a, IL-2, TNFα, and IFNγ. Samples were collected at timepoints D1 (baseline), D63 (1 week PD2), and D175 (1 week PD3). Black lines indicate frequencies in individual samples; blue or red lines the mean frequencies across all samples. D, day; gB, glycoprotein B; HCMV, human cytomegalovirus; IFNγ, interferon gamma; IL, interleukin; PD, post-dose; TNFα, tumor necrosis factor alpha.


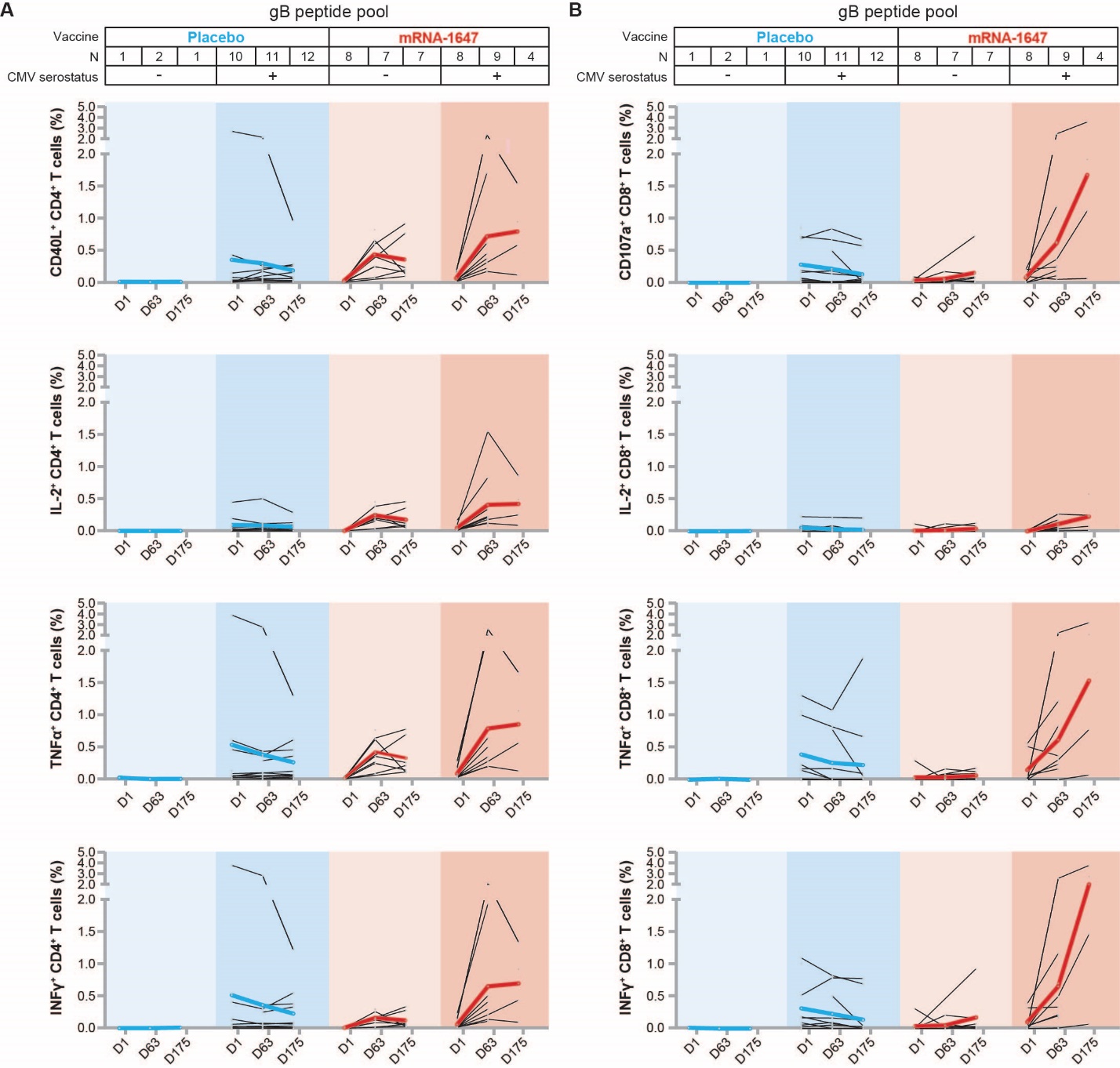


## Supplementary Fig 4. T-cell response in seronegative and seropositive participants who received mRNA-1647 or placebo to stimulation with gL-UL128-UL130-UL131A peptide pool. (A) Frequencies of different CD4^+^ T-cell populations after stimulation with gL-UL128-UL130-UL131A peptide pool. The following markers were measured: CD40L, IL-2, TNFα, and IFNγ. (B) Frequencies of CD8^+^ T cells after stimulation with gL-UL128-UL130-UL131A peptide pool. The following effector cell markers were measured: CD107a, IL-2, TNFα, and IFNγ. Samples were collected at timepoints D1 (baseline), D63 (1 week PD2), and D175 (1 week PD3). Black lines indicate frequencies in individual samples; blue or red lines the mean frequencies across all samples. D, day; gL, glycoprotein L; HCMV, human cytomegalovirus; IFNγ, interferon gamma; IL, interleukin; PD, post-dose; TNFα, tumor necrosis factor alpha.


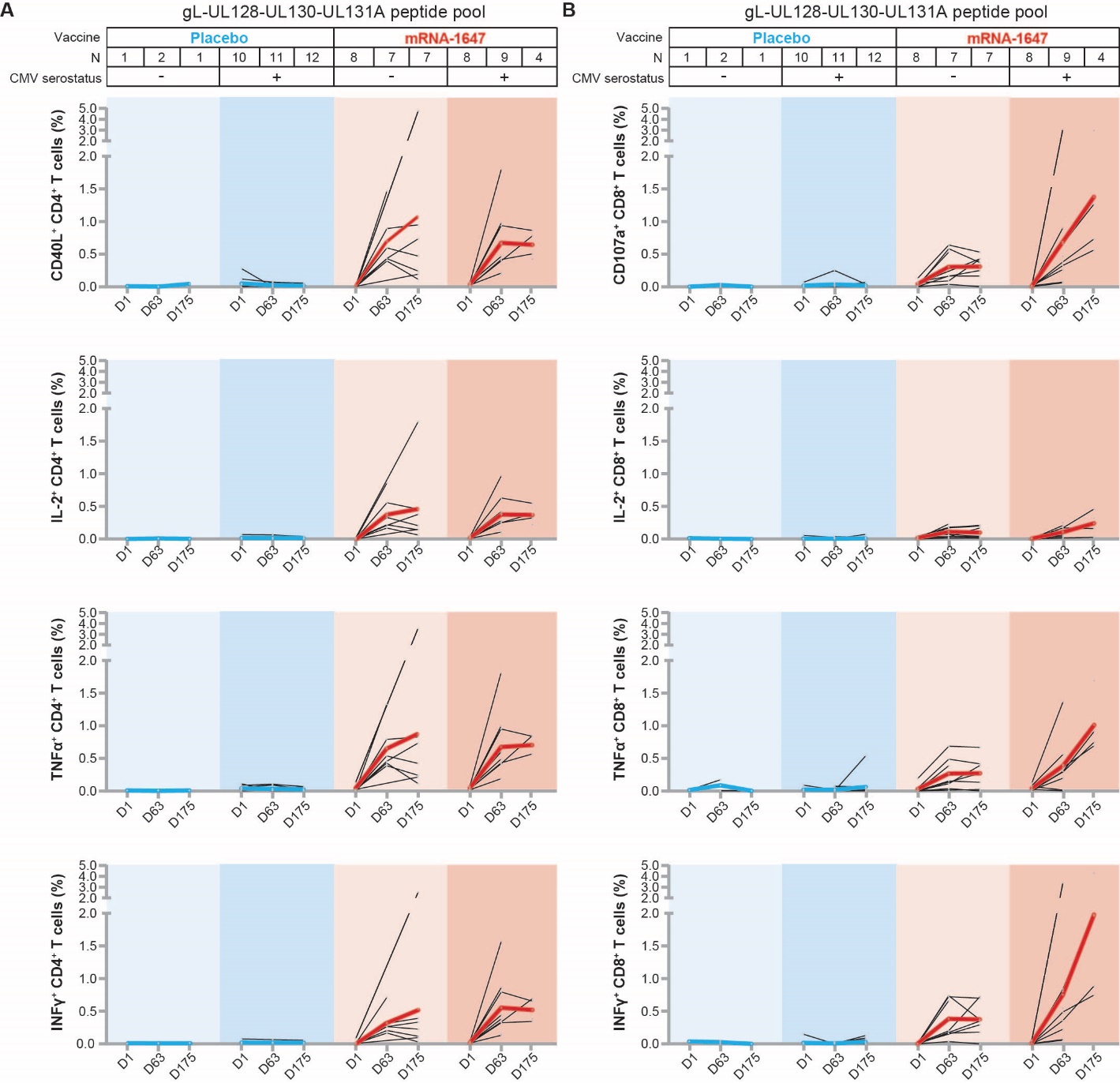


## Supplementary Fig 5. T-cell response in seronegative and seropositive participants who received mRNA-1647 or placebo to stimulation with gH peptide pool. (A) Frequencies of different CD4^+^ T-cell populations after stimulation with gH peptide pool. The following markers were measured: CD40L, IL-2, TNFα, and IFNγ. (B) Frequencies of CD8^+^ T cells after stimulation with gH peptide pool. The following effector cell markers were measured: CD107a, IL-2, TNFα, and IFNγ. Samples were collected at timepoints D1 (baseline), D63 (1 week PD2), and D175 (1 week PD3). Black lines indicate frequencies in individual samples; blue or red lines the mean frequencies across all samples. D, day; gH, glycoprotein H; HCMV, human cytomegalovirus; IFNγ, interferon gamma; IL, interleukin; PD, post-dose; TNFα, tumor necrosis factor alpha.


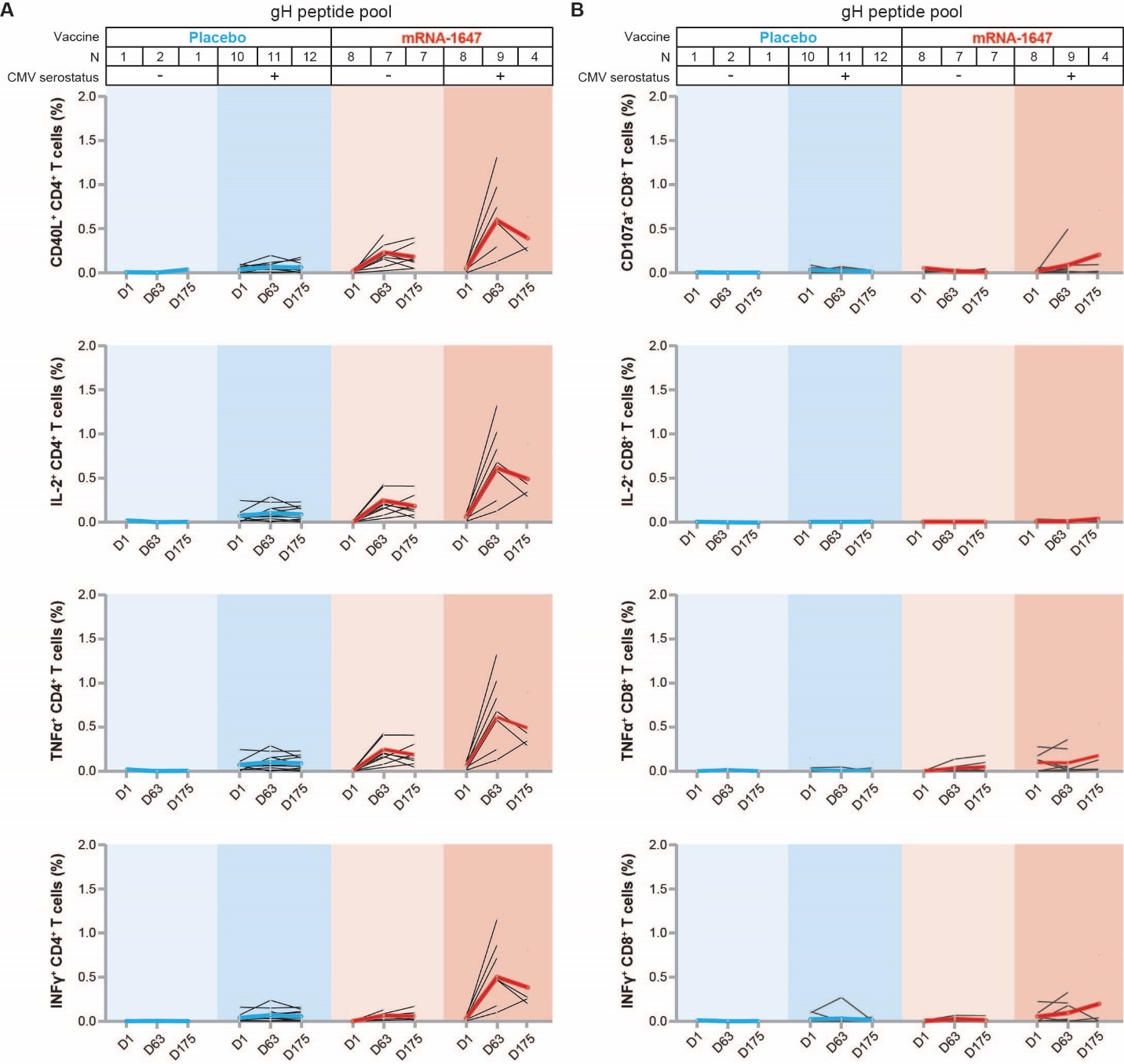


## Supplementary Fig 6. Th2 and Th17 CD4^+^ T-cell response in seronegative and seropositive participants who received mRNA-1647 or placebo. (A) Frequencies of different CD4^+^ T-cell populations after stimulation with gB peptide pool. The following Th2 and Th17 markers were measured:IL-4, IL-5, IL13, and IL-17. (B) Frequencies of CD4^+^ T cells after stimulation with gL-UL128-UL130-UL131A peptide pool. (C) Frequencies of CD4^+^ T cells after stimulation with gH peptide pool. Samples were collected at timepoints D1 (baseline), D63 (1 week PD2), and D175 (1 week PD3). Black lines indicate frequencies in individual samples; blue or red lines the mean frequencies across all samples. D, day; gB, glycoprotein B; gH, glycoprotein H; gL, glycoprotein L; HCMV, human cytomegalovirus; IL, interleukin; PD, post-dose.


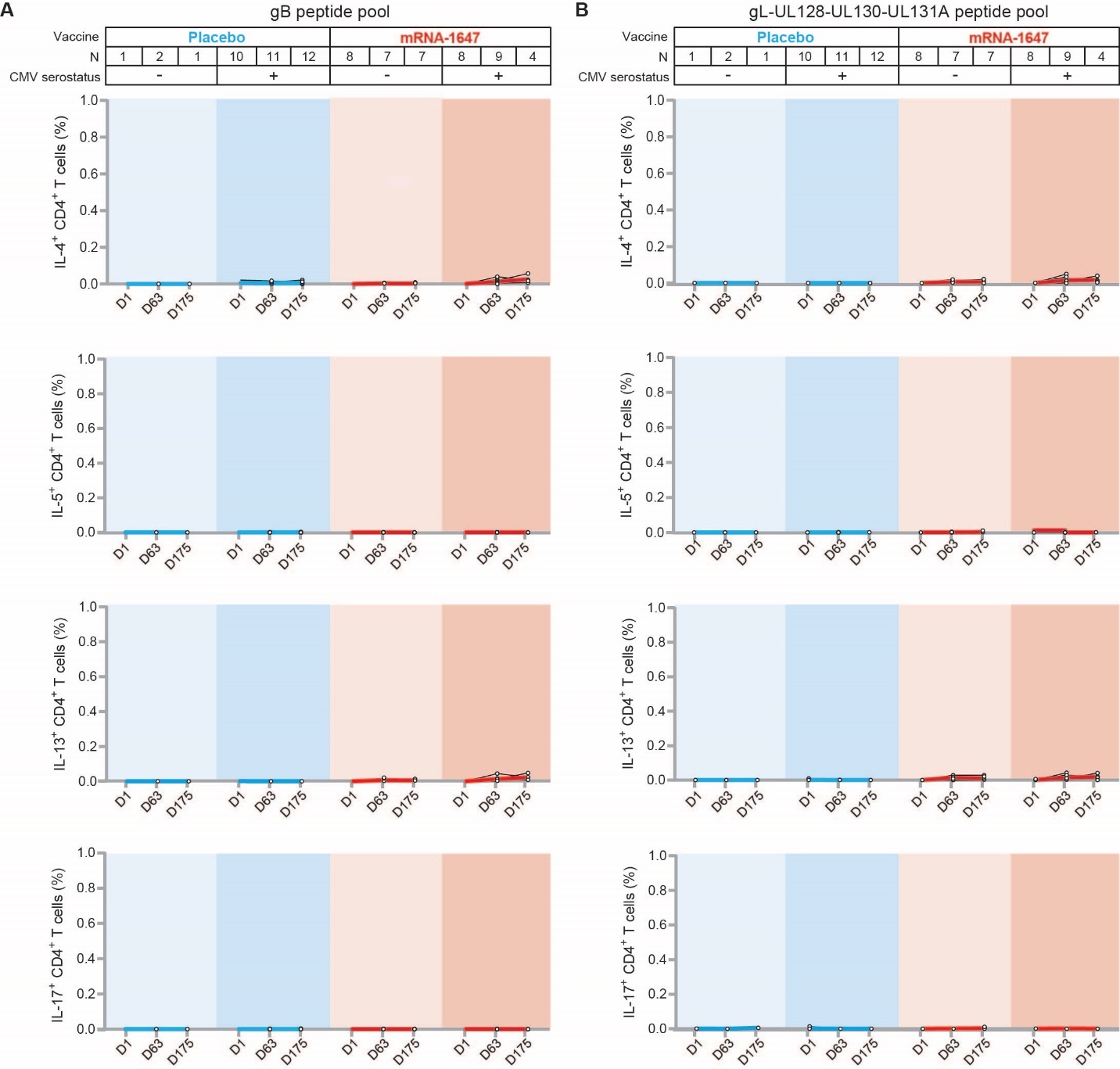


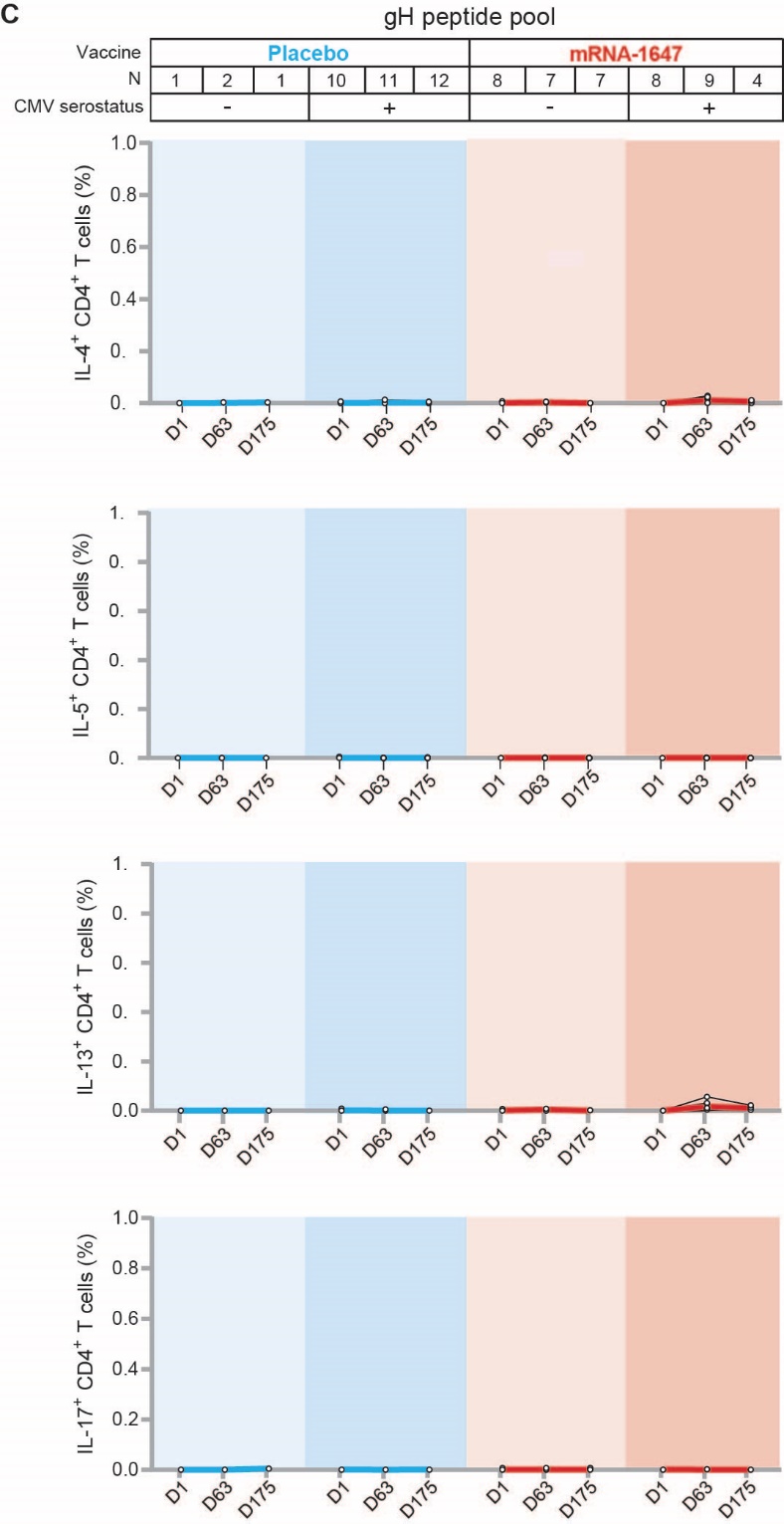


## Supplementary Fig 7. The timeline of immunizations and blood collections for measurements of antibodies, B cells, and T cells. Arrows indicate vaccine/ placebo administration. Red, blue, and orange circles indicate blood draws.

**
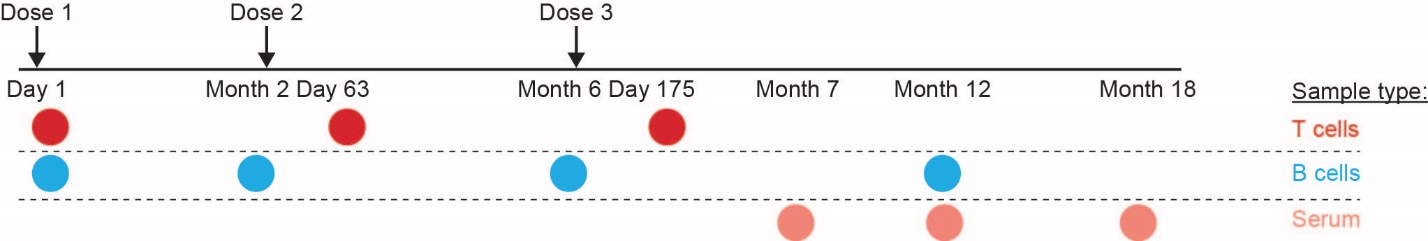
**

## Supplementary Fig 8. Gating strategy used in flow cytometry.


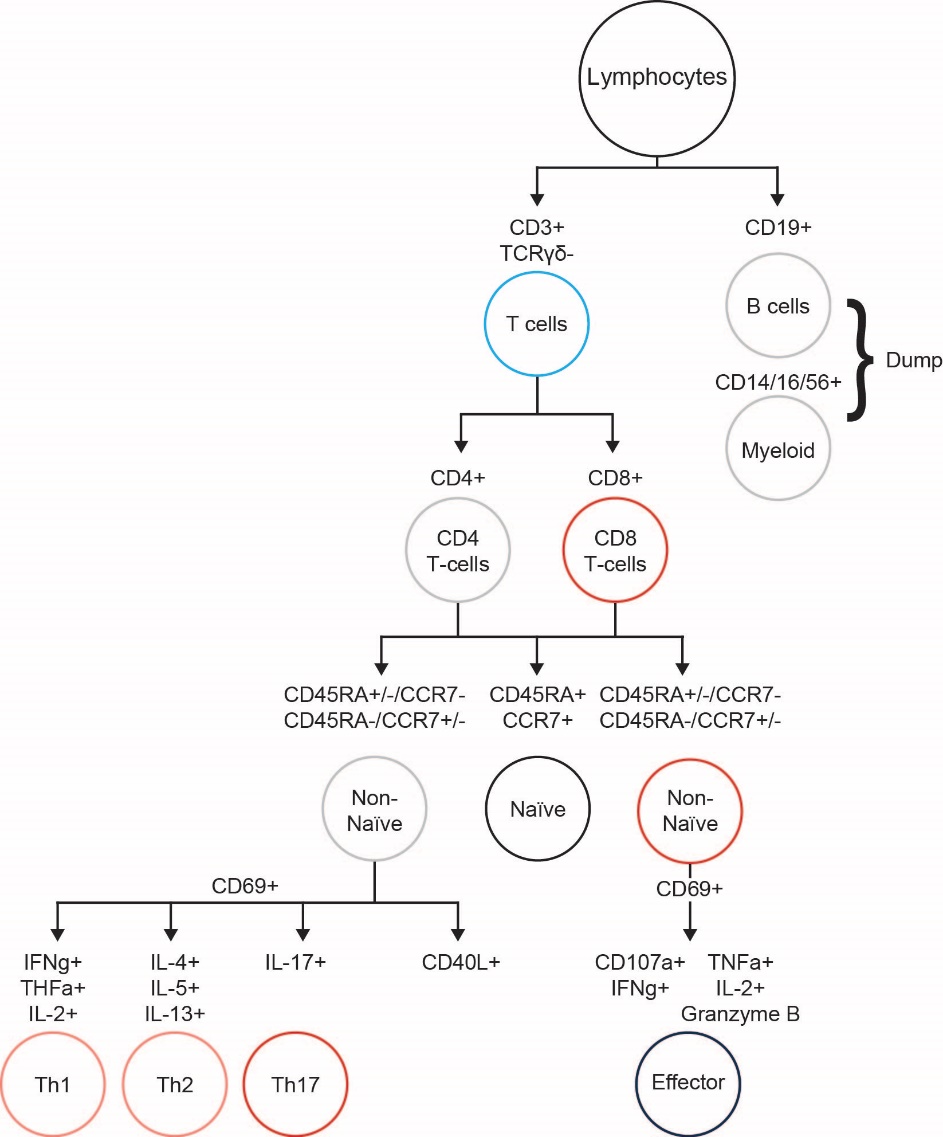

Supplement: Supplemental material — Additional information pertaining to participants and assessments, cells and viruses, etc. [file jvi.01603-23-s0001.docx]
